# Impact of Mean Platelet Volume on Combined Safety Endpoint and Vascular and Bleeding Complications following Percutaneous Transfemoral Transcatheter Aortic Valve Implantation

**DOI:** 10.1155/2013/645265

**Published:** 2013-12-23

**Authors:** Caroline J. Magri, Alaide Chieffo, Alessandro Durante, Azeem Latib, Matteo Montorfano, Francesco Maisano, Michela Cioni, Eustachio Agricola, Remo Daniel Covello, Chiara Gerli, Annalisa Franco, Pietro Spagnolo, Ottavio Alfieri, Antonio Colombo

**Affiliations:** Interventional Cardiology Unit, San Raffaele Scientific Institute, 60 Via Olgettina, 20132 Milan, Italy

## Abstract

*Background*. Vascular and bleeding complications remain important complications in patients undergoing percutaneous transfemoral transcatheter aortic valve implantation (TF-TAVI). Platelets play an important role in bleeding events. Mean platelet volume (MPV) is an indicator of platelet activation. The objective of this study was to assess whether low MPV is an indicator of major vascular and bleeding complications following TF-TAVI. *Methods*. A retrospective cohort study of 330 subjects undergoing TF-TAVI implantation was performed. The primary study endpoint was the occurrence of combined safety endpoint (CSEP); secondary endpoints included major vascular complications and life-threatening bleeding. Endpoints were defined according to Valve Academic Research Consortium 2. *Results*. The CSEP at 30 days was reached in 30.9%; major vascular complications were observed in 14.9% while life-threatening bleeding occurred in 20.6%. Logistic Euroscore and MPV were independent predictors of CSEP. Predictors of vascular complications were female sex, previous myocardial infarction, red blood cell distribution width (RDW), and MPV while predictors of life-threatening bleeding were peripheral arterial disease, RDW, and MPV. *Conclusion*. A low baseline MPV was shown for the first time to be a significant predictor of CSEP, major vascular complications, and life-threatening bleeding following TF-TAVI.

## 1. Introduction

Transcatheter aortic valve implantation (TAVI) has emerged as a valid treatment option for patients with symptomatic severe aortic stenosis and at high risk for conventional surgical aortic valve replacement. This has been reported in multiple national and international registries and the randomized Placement of Aortic Transcatheter Valves (PARTNER) cohort A and B trial [1–8]. The use of next-generation devices combined with increasing expertise in the technique and appropriate patient selection has resulted in encouraging outcomes with regard to both safety and efficacy. However, some worrisome complications do persist. Among these are vascular and bleeding complications which commonly coexist and remain important limitations of TAVI performed via the transfemoral route [5, 9, 10].

The Valve Academic Research Consortium (VARC) has recently revised the VARC definitions and recommendations [11]. The incidence of major vascular complications ranges between 5.6 and 17.3% [7, 8, 12–16]. Such complications not only lead to increased morbidity and impaired quality of life but also to significantly higher 30-day mortality [16]. With regard to bleeding complications, life-threatening bleeding has been reported occurring in 15.6%, whereas any minor, major, or life-threatening bleeding occurred in >40% of patients [17] and, as such, is the most frequent complication occurring post-TAVI. There is currently insufficient data to accurately identify those at highest risk of complications. Novel risk factors are therefore desirable.

Platelets are circulating, anucleate, disc-shaped cells that help to maintain the integrity of blood vessels through adequate haemostasis [18]. Circulating platelets exhibit significant variation in size. Larger platelets contain more granules and produce greater amounts of vasoactive and prothrombotic factors, such as thromboxane A2, serotonin, and adenosine triphosphate (ATP), thus resulting in greater haemostatic efficiency [19, 20]. In fact, increased mean platelet volume (MPV) values are associated with shortened bleeding times [21].

The objective of this study was to assess whether low MPV is an indicator of combined safety endpoint and major vascular and bleeding complications following percutaneous transfemoral aortic valve implantation (TF-TAVI).

## 2. Materials and Methods

### 2.1. Study Design

A nonrandomized retrospective cohort study of all subjects undergoing TAVI in a European institution with established TAVI experience was conducted. Baseline patient characteristics, procedural details, and clinical outcome data from a series of 330 patients who underwent TAVI via the transfemoral approach at San Raffaele Scientific Institute, Milan, Italy, were collected since the introduction of the TAVI program from November 2007 to February 2012. There were no urgent TAVI cases in our series.

**Figure 1:**
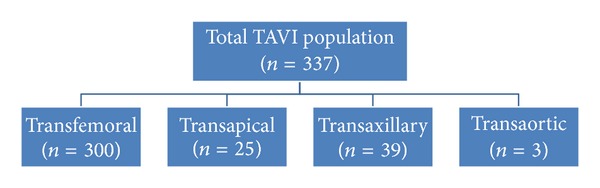
Access sites used in subjects undergoing TAVI from November 2007 to February 2012.

A multidisciplinary Heart Team comprising two interventional cardiologists, a cardiac surgeon, and an anesthesiologist evaluated all patients. The decision to perform TAVI was made in patients with severe symptomatic aortic stenosis with an aortic valve area <1 cm^2^ who were deemed inoperable or at high operative risk by the Heart Team. All patients provided written informed consent for the procedures. In all patients, multimodal imaging was performed to evaluate anatomic suitability for TAVI and determine the optimal access route. The standard access route was the transfemoral (TF) approach; the transapical, transaxillary/subclavian, or transaortic approach was considered whenever the TF approach was found to be inappropriate by the Heart Team. For this analysis, only TF cases were included in view that, in our population, 89% of the cases were done through TF access (Figure 1). In addition there were significant differences in baseline characteristics of the patients doing TAVI via the TF access versus non-TF access. Non-TF access patients had less favorable clinical characteristics with significantly increased incidence of coronary artery disease, chronic obstructive pulmonary disease, and chronic kidney disease, resulting in significantly higher logistic Euroscore and Society of Thoracic Surgeons Predicted Risk of Mortality (STS-PROM). Therefore, in order to have a more homogenous study population and avoid inserting potential confounders in the analysis we focused only on TF patients. Closure strategies were utilized as necessary.

The devices used in this study were the Edwards-SAPIEN/SAPIEN XT (ESV) (Edwards Lifesciences, Irvine, California) and the Medtronic CoreValve ReValving Technology (MCV) (Medtronic Inc., Minneapolis, Minnesota). Use of ESV commenced in November 2007 and MCV was added to our practice in July 2008. The antithrombotic regimen varied slightly interindividually. Generally, patients were loaded with aspirin and clopidogrel (300 mg followed by 75 mg daily) at least 1 day before the intervention. Dual antiplatelet therapy was continued post-TAVI for 1 to 6 months, according to the patient's haematologic parameters, concomitant medications, and associated comorbidities. The antithrombotic regimen was noted for each patient. Furthermore, in all cases, blood samples were drawn in a standard fashion in the fasting overnight state and sent to the laboratory for analysis; blood samples were taken at admission before starting any medication and before performing any intervention. Platelet count, MPV, white cell count, red cell count, haemoglobin, red blood cell distribution width (RDW), and mean corpuscular volume were assessed for all subjects. All measurements were performed at the hospital's central laboratory. MPV measurement was performed by impedance counting using Sismex M2100 Haematologic Analyzer. The normal laboratory reference range for MPV is between 9.1 and 12.5 fL.

The primary study endpoint of the study was the occurrence of combined safety endpoint (CSEP) at 30 days; this comprises all-cause mortality, all types of stroke (disabling and nondisabling), life-threatening bleeding, acute kidney injury (AKI), coronary artery obstruction requiring intervention, major vascular complication, and valve-related dysfunction requiring repeat procedure. Secondary endpoints included major vascular complications and life-threatening bleeding. Endpoints were defined according to VARC 2 [11]. Thus, life-threatening bleeding was defined as (a) fatal bleeding or (b) bleeding in a critical organ, such as intracranial, intraspinal, intraocular, or pericardial necessitating pericardiocentesis or intramuscular with compartment syndrome or (c) bleeding causing hypovolemic shock or severe hypotension requiring vasopressors or surgery or (d) overt source of bleeding with drop in haemoglobin of ≥5 g/dL or whole blood or packed red blood cells (RBCs) transfusion ≥4 units. According to VARC2, major vascular complications include (a) any aortic dissection, aortic rupture, annulus rupture, left ventricle perforation, or new apical aneurysm/pseudoaneurysm or (b) access site or access-related vascular injury (dissection, stenosis, perforation, rupture, arteriovenous fistula, pseudoaneurysm, hematoma, irreversible nerve injury, compartment syndrome, and percutaneous closure device failure) leading to death, life-threatening or major bleeding, visceral ischaemia or neurological impairment or (c) distal embolization (noncerebral) from a vascular source requiring surgery or resulting in amputation or irreversible end-organ damage or (d) the use of unplanned endovascular or surgical intervention associated with death, major bleeding, visceral ischaemia, or neurological impairment or (e) any new ipsilateral lower extremity ischemia documented by patient symptoms, physical exam, and/or decreased or absent blood flow on lower extremity angiogram or (f) surgery for access site-related nerve injury or (g) permanent access site-related nerve injury. Life-threatening bleeding and major vascular events were adjudicated independently by 2 cardiologists and one cardiac surgeon.

All patients provided written informed consent for the procedure and data collection.

### 2.2. Statistical Analysis

All data were analysed using SPSS version 21.0 for Windows. Results are presented as mean ± standard deviation (SD) or median (interquartile range (IQR). Comparisons of continuous variables between two groups were made using independent samples *t*-test for normally distributed variables, while the Mann-Whitney *U* test was used for comparison of nonnormally distributed variables. Categorical variables were compared using the *χ* ^2^ test.

The following covariates were investigated for association with combined safety endpoint at 30 days, major vascular complications, and life-threatening bleeding events following TAVI in univariate analysis: age, sex, body mass index (BMI), body surface area (BSA), logistic EuroSCORE, STS-PROM, previous myocardial infarction (MI), coronary artery bypass grafting (CABG) or percutaneous coronary intervention (PCI), coronary artery disease (CAD), hypertension, chronic obstructive pulmonary disease (COPD), diabetes mellitus, peripheral arterial disease (PAD), chronic kidney disease (i.e., estimated glomerular filtration rate <60 mL/min/1.73 m^2^), cerebrovascular disease, impaired left ventricular (LV) function (ejection fraction <35%), pulmonary hypertension, smoking status, aortic annulus diameter and sheath size, and use of dual antiplatelet agents and proton pump inhibitors. In addition, the following haematological measurements were analyzed: white cell count, haemoglobin, red cell count, red blood cell distribution width, mean corpuscular volume, platelet count, and MPV. MPV was analysed as continuous variable because the cutoff point for MPV with regard to vascular and bleeding complications is not yet established. Significant determinants identified from univariate analysis were studied in a forward stepwise multivariate logistic regression model. Variables were entered into the regression model if their *P* value was <0.1 in univariate analysis. All tests were two sided, and a  *P*  value of  <0.05 was considered to be statistically significant.

The receiver-operator curve analysis was used to define the optimal cutoff value for MPV in predicting freedom from combined safety endpoint, defined as that maximizing the sum of sensitivity and specificity.

## 3. Results and Discussion

### 3.1. Study Population

The baseline characteristics of the overall study population are outlined in Table 1. In total, 330 subjects were assessed with a median age of 81 years and a median logistic Euroscore of 18.54. There was an equal distribution of male and female patients. Thirty-nine per cent of the patients suffered from CAD, 23% had PAD, 29% had diabetes mellitus, 30.5% had chronic renal insufficiency, and 30.7% suffered from COPD while 15% exhibited impaired LV function. The vast majority of the subjects studied were on both dual antiplatelet agents and on proton pump inhibitors. Fifty-eight subjects were taking warfarin prior to the procedure. Coagulation times were within normal limits in patients not taking warfarin. In subjects taking warfarin, the target INR (international normalized ratio) was <1.8 prior to the procedure; this was achieved by stopping warfarin a minimum of 3 days prior to the procedure and giving vitamin K antagonists as required.

### 3.2. Procedural Outcomes

During the study period, 123 MCV and 207 ESV were implanted. The majority of the valves implanted were 26 mm in size (*n* = 162), followed by 23 mm (*n* = 89), 29 mm (*n* = 75), and 31 mm (*n* = 4). With regard to sheaths used, 18 French (Fr) sheaths were used most commonly (*n* = 176), followed by 19 Fr sheaths (*n* = 69), 24 Fr sheaths (*n* = 36), 22 Fr sheaths (*n* = 31), and 15 Fr sheaths (*n* = 17) while a 28 Fr sheath was used in 1 patient. Elective percutaneous closure of the access site was performed in 318 (96.4%) using Prostar percutaneous vascular closure system, with elective surgical closure being performed in the remaining 12 patients. Failure of percutaneous closure was observed in 27 patients (8.2%).

Device success was achieved in 306 patients (92.7%). The incidence of periprocedural complications was relatively low. Thus, valve embolization occurred in 11 patients (3.3%) while valve recapturing was performed in 7 patients (2.1%). A second valve was necessary in 12 patients (3.6%). Aortic dissection was observed in 2 patients (0.6%). Cardiac tamponade occurred in 13 patients (3.9%), shock was seen in 26 patients (7.9%), and urgent cardiothoracic surgery was necessary in 4 patients (1.2%). With regard to in-hospital outcomes, 6 patients (1.8%) suffered from a transient ischaemic attack and 3 patients (0.9%) suffered from a stroke. Similarly, in-hospital MI was noted in 3 patients while 13 patients (3.9%) died during their hospital stay. Renal replacement therapy was required in 18 patients (5.3%) while a permanent pacemaker was implanted post-TAVI in 50 patients (13.2%).

In our study cohort, the CSEP was reached in 30.9% (*n* = 102 patients). Life-threatening bleeding (seen in 67 patients, i.e., 20.3%) and major vascular complications (seen in 49 patients, i.e., 14.8%) contributed mainly to CSEP. With regard to the other components, all-cause mortality was seen in 13 patients, stroke in 3 patients, AKI in 27 patients, and coronary artery obstruction in 1 patient while valve-related dysfunction requiring repeat procedure occurred in 10 patients.

The significant predictors of the outcomes are reported in Table 2. MPV was shown to be a predictor of both the primary and secondary endpoints. Thus, logistic Euroscore and MPV were independent predictors of CSEP. With regard to major vascular complications, female sex, previous MI, RDW, and MPV were shown to be significant determinants in multivariate analysis while PAD, RDW, and MPV were shown to be independent predictors of life-threatening bleeding. No significant associations were shown between the outcomes studied, BMI, and STS-PROM. Also, dual antiplatelet administration, warfarin utilization, baseline haemoglobin levels, and baseline platelet count levels were not found to be independent predictors of the outcomes studied.

Univariate analysis was also performed to assess whether MPV was associated with all-cause and cardiac mortality; however, it was not found to be statistically significant (*P* = 0.17 and *P* = 0.37, resp.).

**Figure 2:**
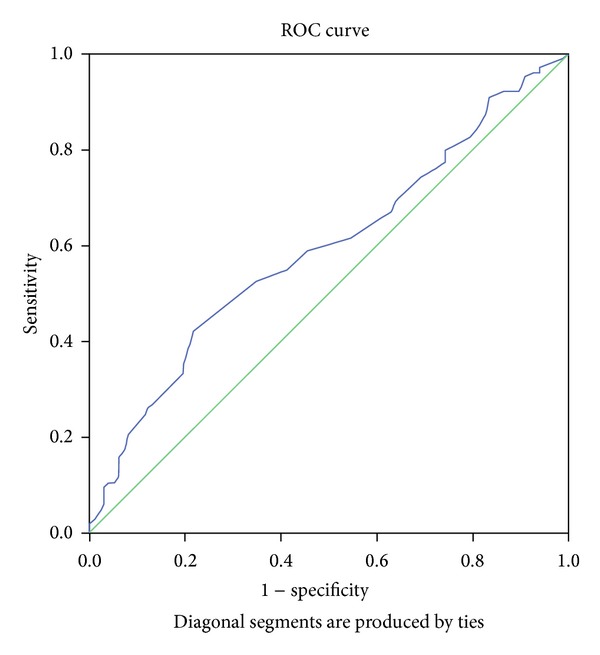
The receiver-operating characteristic (ROC) curve for MPV for predicting combined safety endpoint at 30 days (area under curve 0.59, 95% CI 0.53–0.66, *P* = 0.008).

Post-hoc receiver-operating characteristic (ROC) curve analysis was consequently performed to further analyze the relation between MPV and combined safety endpoint. An MPV value of 10.75fL was found to have 58% sensitivity and 54% specificity for predicting freedom from combined safety endpoint (area under curve 0.59, 95% CI 0.53–0.66, *P* = 0.008) (see Figure 2).

### 3.3. Discussion

The main findings of this study were as follows.

1. Baseline MPV is an independent predictor of CSEP at thirty days, as well as major vascular complications and life-threatening bleeding events following TF-TAVI. Such an association is independent of usage of dual antiplatelet agents, baseline haemoglobin, and baseline platelet levels.
2. A cutoff point for MPV of 10.75fL is identified. Above this value a lower incidence of vascular and bleeding complications is observed, resulting in lower CSEP.

Despite the gradual decrease in sheath and catheter size and increasing expertise, vascular and bleeding complications remain significant pitfalls of current TAVI devices. In a meta-analysis of 16 studies including 3,519 patients, life-threatening bleeding occurred in 15.6% (95% CI: 11.7% to 20.7%) while major vascular complications occurred in 11.9% (95% CI: 8.6% to 16.4%) [17]. Khatri et al. have recently performed a meta-analysis comprising 49 studies which enrolled 16,063 patients and showed that vascular complications are a significant complication of transarterial procedures compared with transapical procedures (14.2% versus 3.4%; *P* < 0.001) [22]. This is comparable to the major vascular complication rate seen in our study population. A decrease in these complications is desirable as this would translate in reduced morbidity and mortality in the frail TAVI population, as demonstrated in a pooled analysis of five studies including >1,000 patients whereby 16.9% of patients with vascular access complications died at 30 days versus 6.6% in those without (OR 2.88, 95% CI 1.71–4.86) [23].

To the best of our knowledge, this is the first study to report the correlation between MPV and CSEP mostly due to the impact on vascular and bleeding complications in patients undergoing TF-TAVI. With regard to the role of MPV in vascular complications, it is reasonable to assume that a subject who suffers from some form of platelet dysfunction is more likely to develop haematoma, percutaneous closure device failure, and life-threatening or major bleeding, all of which are included in the definition of major vascular complications. Analyses on both bleeding and vascular complications have been performed to indicate further the importance of MPV in subjects undergoing TF-TAVI. MPV is an inexpensive and readily available haematologic parameter that is routinely reported with complete blood count and consequently could serve as a useful adjunctive tool in identifying subjects who are at high risk of bleeding following TF-TAVI.

Apart from the platelet count, MPV is the most commonly measured platelet index. Studies have indicated that MPV is an indicator of platelet reactivity [19, 24, 25]. Larger platelets are denser and contain more alpha granules, dense granules, and lysosomes, which release prothrombotic factors [26–28]. They exhibit increased thromboxane A2 biosynthesis and express a greater number of adhesion molecules, such as P-selectin and glycoprotein (GP) IIb/IIIa [29, 30]. Larger platelets also demonstrate greater aggregability in response to ADP and decreased inhibition of aggregation by prostacyclin in vitro [31, 32], thus leading to increased thrombotic activity. Interestingly, a number of studies have shown that MPV is associated with cardiovascular risk factors such as obesity, diabetes mellitus, and metabolic syndrome, is predictive of stroke, acute myocardial infarction (AMI), and restenosis following coronary angioplasty, and is a bad prognosticator in patients with stroke and AMI (reviewed by Vizioli et al. [33]). With regard to the association between low MPV and bleeding events, Makay et al. showed that MPV was significantly lower in Henoch-Schönlein purpura patients with gastrointestinal bleeding than patients without bleeding [34].

Interestingly, platelet dysfunction has been implicated in patients with aortic stenosis (AS). Vincentelli et al. demonstrated that the association between aortic stenosis and gastrointestinal bleeding is secondary to a reduction in the proportion of high-molecular-weight von Willebrand factor (vWF) multimers and such impaired distribution of the multimers could be restored following valve replacement [35]. Large vWF multimers are stored within endothelium (in Weibel-Palade bodies) and platelets (*α*-granules). It is possible that there is a decrease in platelet size with increasing shear stress present in aortic stenosis, resulting in low MPV indices. The latter could be indicative of a decrease in large vWF multimers secondary to a decrease in platelet *α*-granules. Alternatively, platelets with low MPV exhibit abnormal platelet adhesion. In fact, the interaction between vWF and platelet GP Ib plays a critical role in the initial phase of platelet adhesion, particularly under high shear stress [36]. This is in keeping with the reduction in platelet retention rates exhibited in almost all patients with severe AS [37]. Interestingly, our group has recently shown an abnormally high activated clotting time in subjects undergoing TAVI [38]; this could also be an indicator of platelet dysfunction which is possibly vWF-mediated and contributes to bleeding tendency in patients with severe AS.

CSEP was chosen as the primary endpoint in view of bigger number of events and thus more significant results are obtained. Life-threatening bleeding and major vascular complications were the two major contributing factors to CSEP. Significant predictors of CSEP were MPV and logistic Euroscore. The latter is expected, being an indicator of the patient's comorbidities and frailty state. With regard to MPV, a low MPV was independently associated with increased vascular and bleeding complications, these being the major determinants of CSEP in our TAVI cohort. In fact MPV, RDW, female sex, and past history of MI were significant predictors of major vascular complications. These findings are consistent with the results from the Paris-Rotterdam-Milano-Toulouse in Collaboration (PRAGMATIC) study [39] whereby female sex and sheath size were independent predictors for major vascular complications. Similarly, Buchanan et al. had shown a trend towards increased major vascular complications in the female sex [40]. Interestingly, while sheath size was significant in univariate analysis, it lost its significance on performing multivariate logistic regression analysis, indicating that MPV is a stronger predictor of vascular complications. In addition, the vast majority of the procedures were performed with an 18 or 19 Fr sheath. A beneficial effect of smaller sheaths has been shown in TF-TAVI [39, 41, 42]. With regard to the association with history of MI, this is probably secondary to the presence of severe and diffuse atherosclerosis that predisposes these patients to major vascular complications.

Peripheral arterial disease, RDW, and MPV levels were shown to be independent predictors of life-threatening bleeding. The association between this secondary outcome and MPV is weaker compared to the association with the other outcomes studied; this is probably secondary to the low number of life-threatening bleeding events in our patient cohort. Alternatively, this could be due to other variables being included in the model which have an effect on the multivariate analysis. The association between PAD and bleeding events is probably secondary to difficult vascular access in patients with PAD, resulting in more bleeding. This is in line with the current data on TAVI patients whereby the presence of PAD has been shown to be a predictor not only of major vascular complications but also of mortality in patients with percutaneous and surgical TAVI [43]. In addition, PAD is also a marker of generalized atherosclerosis, which is associated with weakening of the arterial wall. As regards RDW, it is another novel haematologic parameter and a measure of heterogeneity in erythrocyte size reported as part of a standard complete blood count. It has gained increasing importance in recent years as an independent prognosticator of adverse outcomes in cardiovascular disease, as demonstrated in various studies. It has been speculated that RDW is an integrative measure of underlying pathologic conditions leading to increased oxidative stress and subclinical inflammation; however, further studies are needed.

The novel association between low MPV and vascular/bleeding complications in patients undergoing TAVI might assist in the choice of antiplatelet agents administered as well as selection of access route. The current recommendation to perform TAVI under a combination of heparin, aspirin, and clopidogrel is merely empiric and further studies are needed to identify the ideal choice of antiplatelet agent. In view of our study, it would be interesting to investigate whether the choice of antiplatelet treatment can be guided by MPV levels. Furthermore, in view of the mounting experience with alternative access routes (transaxillary/subclavian, transaortic, and transapical), multimodal imaging assessment of the arterial tree may improve TAVI access strategy selection [44].

Our study has several limitations. It is a nonrandomized retrospective study. It was not possible to assess inter-operator variability due to the high correlation with variables entered in the analysis. The learning curve effect was also not accounted for. However, in the PRAGMATIC study, a learning curve effect was not identified as an independent predictor of access site complications, possibly secondary to the high technical skills of the operators with other complex catheter-based procedures before embarking on a TAVI program. Another limitation is lack of quantitative analysis of peripheral arterial vasculature such as arterial vessel size, sheath-femoral artery ratio (SFAR), calcification, and tortuosity, which could be potential interfering variables. Also we lack information on frailty status and platelet aggregation studies. However, we were interested in identifying prognostic markers which can be easily measured in clinical practice rather than those which are only available in a research setting. In addition, access strategy was determined by expert operators, based on clinical judgment and qualitative interpretation of available imaging procedures. Furthermore, we managed to adjust for multiple potential confounders that could influence the combined safety endpoint including sheath size and antiplatelet therapy at baseline. We thus believe that the study population is representative of daily life patients and adequately reflects contemporary TAVI practice.

Further studies of larger patient populations are required to confirm our results, especially the cutoff point for MPV of 10.75 fL identified using ROC analysis.

## 4. Conclusions

In spite of the increasing expertise and technological advancements, vascular and bleeding complications after TF-TAVI are still present. A low baseline MPV was shown for the first time to be a significant predictor of CSEP, major vascular complications, and life-threatening bleeding in subjects undergoing TF-TAVI.

## Figures and Tables

**Table 1:** Baseline and procedural characteristics of study population.

Patient characteristics (*n* = 330)	Values
Age, years^†^	81 (76–85)
Male : female (*n*)	165 : 165
Body mass index, (kg/m^2∗^)	26.43 ± 4.81
Body surface area, (m^2∗^)	1.78 ± 0.18
Previous myocardial infarction (*n* (%))	62 (18.8%)
Previous percutaneous coronary intervention (*n* (%))	62 (18.8%)
Previous coronary artery Bypass Grafting (*n* (%))	63 (19.1%)
Coronary artery disease (*n* (%))	125 (38.8%)
Diabetes mellitus (*n* (%))	95 (28.9%)
Current smoker (*n* (%))	10 (3.0%)
Hypertension (*n* (%))	244 (74.2%)
Hyperlipidaemia (*n* (%))	194 (59.0%)
Left ventricular ejection fraction <35% (*n* (%))	48 (14.7%)
Cerebrovascular disease (*n* (%))	41 (12.5%)
Chronic kidney disease (*n* (%))	100 (30.5%)
Pulmonary hypertension (*n* (%))	56 (17.4%)
Chronic obstructive airways disease (*n* (%))	101 (30.7%)
Peripheral arterial disease (*n* (%))	75 (22.9%)
Logistic Euroscore^†^	18.54 (11.16–30.57)
STS-PROM^†^	5.5 (3.6–9.1)
Antiplatelet use (none : single : dual) (*n*)	37 : 86 : 206
Proton-pump inhibitor use (*n* (%))	254 (81.2%)
Aortic annulus diameter, (mm^†^)	24 (22–25)
Sheath size, (mm^†^)	18 (18-19)
White cell count, (×10^9^/L^†^)	6.55 (5.33–8.0)
Red cell count, (×10^12^/L*)	4.23 ± 0.59
Haemoglobin, (g/dL*)	12.17 ± 1.72
Mean corpuscular volume, (fL^†^)	89.55 (89.83–93.30)
Red blood cell distribution width, (%^†^)	14.7 (13.8–16.0)
Platelet count, (×10^9^/L*)	201.43 ± 72.37
Mean platelet volume, (fL*)	10.94 ± 1.13

Values are expressed as mean ± SD* or median (IQR)^†^ or number (% of patients).

STS-PROM: Society of Thoracic Surgeons Predicted Risk of Mortality.

**Table:** (a)

Variable	Univariable *P* value	Multivariable odds ratio (95% CI)	*P* value
Logistic Euroscore	0.004	1.02 (1.01–1.04)	0.002
Chronic kidney disease	0.014		
MPV (fL)	0.016	0.77 (0.62–0.97)	0.024
RDW (%)	0.004		

**Table:** (b)

Variable	Univariable *P* value	Multivariable odds ratio (95% CI)	*P* value
Female gender	0.045	2.54 (1.26–5.14)	0.009
Previous MI	0.005	3.25 (1.56–6.76)	0.002
BSA (m^2^)	0.031		
Sheath size (Fr)	0.038		
MPV (fL)	0.014	0.7 (0.50–0.96)	0.028
RDW (%)	0.027	1.16 (1.01–1.32)	0.035

**Table:** (c)

Variable	Univariable *P* value	Multivariable odds ratio (95% CI)	*P* value
Peripheral arterial disease	0.008	2.40 (1.25–4.59)	0.008
BSA (m^2^)	0.053		
Logistic Euroscore	0.026		
Aortic annulus diameter (mm)	0.024		
MPV (fL)	0.015	0.76 (0.58–1.00)	0.05
RDW (%)	0.004	1.15 (1.01–1.32)	0.036

BSA: body surface area; CSEP: combined safety endpoint; MI: myocardial infarction; MPV: mean platelet volume; PCI: percutaneous coronary intervention; RDW: red blood cell distribution width; VASC: vascular complications.
